# The Effect of Hydroxychloroquine on Residual Proteinuria in Patients With Immunoglobulin A Nephropathy: A Retrospective Study Based on Propensity Score Matching

**DOI:** 10.3389/fmed.2022.922365

**Published:** 2022-07-06

**Authors:** Mijia Liu, Xueyan Bian, Li Wang, Guisen Li

**Affiliations:** ^1^Department of Nephrology, Institute of Nephrology, Sichuan Academy of Medical Sciences and Sichuan Provincial People’s Hospital, School of Medicine, University of Electronic Science and Technology of China, Sichuan Clinical Research Center for Kidney Diseases, Chengdu, China; ^2^Department of Nephrology, Institute of Nephrology, Ningbo First Hospital, Ningbo Hospital of Zhejiang University, Ningbo, China

**Keywords:** IgA nephropathy, hydroxychloroquine, residual proteinuria, corticosteroids, immunosuppressives

## Abstract

**Background:**

There is insufficient evidence to support the use of hydroxychloroquine (HCQ) in Immunoglobulin A nephropathy (IgAN) patients with high residual proteinuria in spite of 6-month supportive treatment combined with corticosteroids (P) and/or immunosuppressives (IM). This study aims to explore the effect of HCQ on residual proteinuria in IgAN.

**Materials and Methods:**

This is a retrospective study. IgAN patients who had residual proteinuria ≥0.3 g/24 h after 6-month treatment by renin-angiotensin system inhibitors (RASI) + P ± IM were included. Groups were divided based on the different regimens and then matched by the propensity score matching method. The primary outcome was defined as the cumulative frequency of residual proteinuria reduction ≥30%.

**Results:**

RASI (*n* = 183), HCQ + RASI (*n* = 59), RASI + P ± IM (*n* = 145), and HCQ + RASI + P ± IM (*n* = 38) groups were included. HCQ + RASI group had a higher level of residual proteinuria and a worse renal function than those in the RASI group. The renal function was worse in the HCQ + RASI + P ± IM group than that in the control group, but residual proteinuria levels were similar. After matching, there were 40 patients in the first two groups and 29 patients in the latter two groups, respectively. The cumulative frequency of residual proteinuria reduction ≥30% in HCQ + RASI + P ± IM group was higher than that in control group (86.2% vs. 62.1%, χ^2^ = 6.397, *p* = 0.011). HCQ combination treatment was one of independent factors.

**Conclusion:**

The addition of HCQ treatment can effectively reduce the residual proteinuria in IgAN patients previously treated with supportive treatment combined with P and IM treatment and the cumulative frequency of effective reduction of residual proteinuria can reach 86.2%.

## Introduction

Immunoglobulin A nephropathy (IgAN) is the most common primary glomerular disease in the world (1, 2). Proteinuria is the strongest predictor of ESKD and persistent proteinuria remission can reduce the risk of IgAN progression, even for a short period (3–5). The guidelines of kidney disease: improving global outcomes (KDIGO) and previous studies proposed that proteinuria can be used as an alternative outcome indicator and reducing proteinuria is one of the targets of IgAN treatment (6–9). A meta-analysis reported that patients with lower proteinuria levels had a lower risk of renal composite endpoints (10). However, IgAN patients with proteinuria <1 g/24 h may not have a good prognosis. A prospective study showed that the incidence of chronic renal failure and ESKD in patients with proteinuria <1 g/24 h were 35.3 and 16.5%, respectively (11). Shen found that proteinuria index (defined as the product of proteinuria and course of the disease at the time of renal biopsy) was related to renal prognosis and can be used as a marker to predict the progress of IgAN (12). Reich found that patients with origin proteinuria >3 g/24 h whose proteinuria decreased below 1 g/24 h had a similar prognosis to those with proteinuria <1 g/24 h and were better than those who had never achieved remission (5).

In previous studies and the guideline of KDIGO, proteinuria remission was defined as 24-urinary protein <0.3 g/24 h (3, 6). In addition, we previously found that IgAN patients didn’t reach proteinuria remission (<0.3 g/24 h) and the residual proteinuria was even more than 3 g/24 h for a long time, despite receiving the optimized supportive treatment combined with P and IM therapy (13). For these populations, clinicians often use P or IM for a longer time or larger dose to further reduce proteinuria. Although it is beneficial to some patients, the systemic adverse reactions caused by the long-term use of P or IM can’t be ignored.

Hydroxychloroquine (HCQ), as an immunomodulator, is mainly used in autoimmune diseases and empirically used as an alternative treatment for corticosteroid-intolerant or –contraindicated patients. *In vivo* and *in vitro* experiments, it is reported that HCQ can reduce the production of pathogenic IgA (14, 15). Moreover, clinical studies have found that HCQ can reduce proteinuria in IgAN patients (16–18). A randomized controlled trial found that HCQ could reduce proteinuria during the short treatment period (19). Recently, Tang reported the long-term efficacy and safety of HCQ, supporting HCQ could be used as a supportive treatment for IgAN (20). The KDIGO 2021 Clinical Practice Guideline for IgAN proposed that HCQ can be used as an alternative or supplement to P and IM therapy (6).

However, there is insufficient evidence to support the use of HCQ in IgAN patients with residual proteinuria. Based on this, we focus on the impact of HCQ on residual proteinuria in IgAN patients after at least 6 months of supportive treatment combined with P and IM treatment, evaluating the clinical efficacy and safety of HCQ treatment and providing a new scheme of sequential treatment of IgAN.

## Materials and Methods

### Study Design

#### Patients

This was a retrospective case-control study performed in two centers. 1225 cases in Sichuan Provincial People’s Hospital from January 2007 to August 2019 and 519 cases in Ningbo First Hospital from January 2012 to January 2019 were reviewed, respectively. Then, 674 patients with residual proteinuria ≥ 0.3 g/24 h after receiving supportive treatment combined with P or IM treatment for at least 6 months were screened. This study was approved by the Ethics Committees of Sichuan Provincial People’ Hospital and the Ethics Committees of Ningbo First Hospital, and informed consent was obtained before the study. We had access to information that could identify individual participants during collection.

#### Inclusion and Exclusion Criteria

The inclusion criteria were as follows: (1) Primary IgAN diagnosed by renal biopsy; (2) Residual proteinuria ≥0.3 g/24 h after receiving RASI + P ± IM regimen for at least 6 months; (3) Followed up for at least 6 months; (4) All patients signed informed consent forms. The exclusion criteria included secondary IgAN, missing clinicopathological data at biopsy, complicated with Henoch-Schönlein nephritis, chronic hepatic disease, malignant tumor, systemic lupus erythematosus or other connective tissue diseases, etc., using RASI + P ± IM less than 6 months, complete remission, followed up less than 6 months and using HCQ only during the follow-up period.

#### Data Acquisition

The patients’ clinicopathologic characteristics at biopsy and follow-up data were collected, including age, sex, body mass index (BMI), mean arterial pressure (MAP), 24-hour urine protein (24-h Upro), serum creatinine (Scr), estimated glomerular filtration rate (eGFR), blood urea nitrogen (BUN), uric acid (UA), hemoglobin (Hb), albumin (Alb), pathological data [MEST-C scores based on the Oxford classification of IgAN (21)]. The treatment regimens and the adverse drug reactions were collected.

#### Treatment Protocol

The ophthalmologic examination of retinal, macular and visual field lesions was conducted before HCQ treatment. After use of HCQ, patients performed retinal evaluations every 3–6 months. HCQ was given to IgAN patients as follows: when eGFR >45 ml/min/1.73 m^2^, the oral dose of HCQ was 200 mg twice a day; When eGFR is 30 ∼ 45 ml/min/1.73 m^2^, the oral dose of HCQ is 100 mg, twice or three times a day; eGFR was <30 ml/min/1.73 m^2^, and the oral dose of HCQ was 100 mg once a day. According to the KDIGO guidelines (6), RASI, P and IM were given as follows: (1) Supportive treatments such as reducing blood pressure, using RASI drugs, etc. (2) Patients with urinary protein >0.5 g/24 h were initially given RASI and maximal supportive care was given depending on the blood pressure. (3) Patients at high risk of progressive IgAN were given corticosteroids and strictly followed the absolute or relative contraindications. (4) For patients with proteinuria >1 g/24 h, prednisone 0.6–1.0 mg/kg/d for 4–8 weeks, followed by slow reduction, and the total course of treatment is 6–12 months. (5) For rapidly progressing crescentic IgAN patients or patients with proteinuria >1 g/24 h who glucocorticoids intolerance or contraindication, or for patients with severe proteinuria and pathological manifestations, P and IM agents can be used. (6) The usage and dosage of IM are used based on the different levels of eGFR and the experience of clinicians, including cyclophosphamide, azathioprine, cyclosporine, tacrolimus, mycophenolate mofetil, cyclosporine A, leflunomide, tripterygium glycosides, etc.

#### Case Grouping and Matching

Based on different treatment regimens and whether combined with HCQ, eligible patients were divided into the RASI group, HCQ + RASI group, RASI + P ± IM group, and HCQ + RASI + P ± IM group. All four groups were observed and analyzed to determine factors with different distributions, respectively. Factors with different distributions, confounding factors and offset factors were included for matching and then 1:1 propensity score matching (PSM) (nearest neighbor matching method) was used by the MatchIt package based on R software. The caliper’s value was 0.035.

### Definitions

Body mass index was calculated as BMI = weight/height^2^. MAP was calculated as 1/3 systolic blood pressure + 2/3 diastolic blood pressure. eGFR was calculated by the Chronic Kidney Disease Epidemiology Collaboration (CKD-EPI) creatinine equation (22). Δ24 h Upro was calculated as the difference between the 24-h urine protein quantification at a time point during the follow-up period and the time of enrollment. The proteinuria remission rate was calculated as the proportion of patients with partial remission and complete remission to total patients. Referring to previous studies (23), complete remission was defined as urinary protein quantification <0.3 g/24 h, and partial remission was defined as urinary protein excretion ≥0.3 g/24 h but decreased by ≥50% compared with baseline. Residual proteinuria was defined as 24-h urinary protein ≥0.3 g/24 h after RASI + P ± IM regimen for at least 6 months. The effective reduction rate of residual proteinuria was defined as residual proteinuria reduction ≥30%.

The primary outcome was defined as the cumulative frequency of residual proteinuria reduction ≥30%. The secondary outcome was defined as the change in proteinuria, the change in serum creatinine, the percent changes in proteinuria and the proteinuria remission rate.

### Statistical Analyses

To reduce the bias of the retrospective study, propensity score matching analysis by 1:1 fixed ratio in R using the MatchIt package with nearest-neighbor matching was performed and the caliper value was set to 0.035. Normally distributed data are expressed as mean ± standard deviation and continuous variables were compared by *t*-tests. Non-normally distributed data are expressed as median (Q25, Q75) and continuous variables were compared by Wilcoxon rank-sum tests. Categorical data were presented in percentages and nominal variables were compared by Chi-square tests. The Kaplan–Meier method was used to calculate the cumulative frequency of residual proteinuria reduction ≥30%. Spearman rank was used to analyze the correlation between clinicopathological features and the effective reduction frequency in residual proteinuria. Univariate and multivariate logistic regression models were used to analyze the independent factors associated with residual proteinuria reduction ≥30%. A two-tailed *p* < 0.05 was considered statistically significant.

## Results

### Screening

**FIGURE 1:**
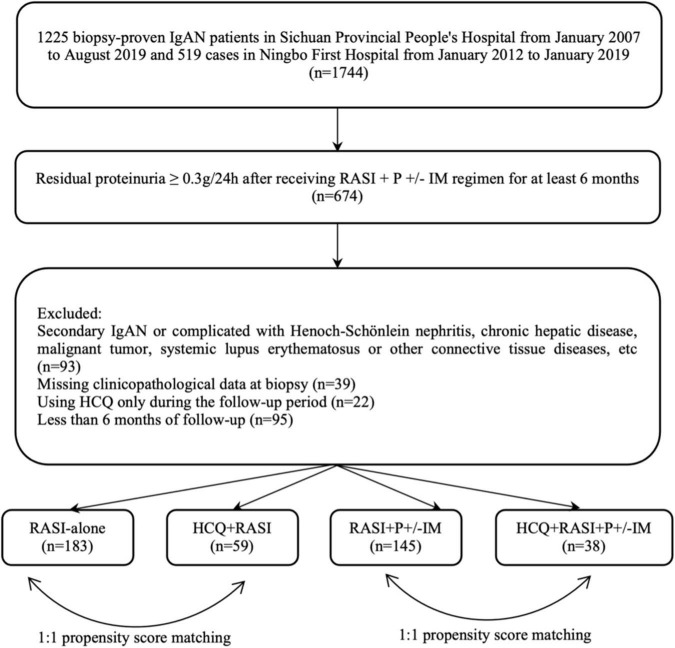
Flow chart for screening IgAN patients with residual proteinuria after at least 6 months of traditional therapy.

A total of 1744 patients with primary IgAN diagnosed by renal biopsy were screened. Among these, 674 patients with 24-h proteinuria ≥0.3 g after optimized supportive treatment combined with P and/or IM for at least 6 months were followed up. Patients with a lack of clinicopathological data at renal biopsy (*n* = 39), secondary IgAN (*n* = 93), use of HCQ only during the follow-up period (*n* = 22) and less than 6 months of follow-up (*n* = 95) were excluded. 425 cases met the inclusion criteria, and the median follow-up time was 6.0 (5.6, 6.4) months. The flow chart is shown in Figure 1.

### Comparison of Clinical and Pathological Data at Renal Biopsy

Based on different treatment regimens and whether combined with HCQ, they were divided into RASI group (*n* = 183), HCQ + RASI group (*n* = 59), RASI + P ± IM group (*n* = 145), and HCQ + RASI + P ± IM group (*n* = 38). There was no significant difference between the RASI group and HCQ + RASI group in sex, age, BMI, MAP, 24-h Upro, Scr and pathology data at renal biopsy, as well as between RASI + P ± IM group and HCQ + RASI + P ± IM group, shown in Table 1. Drug regimens before enrollment were also similar between RASI + P ± IM group and HCQ + RASI + P ± IM group. However, HCQ + RASI group received more corticosteroids before enrollment than RASI group, shown in Table 1.

**TABLE 1:** The clinicopathological characteristics of IgAN patients with residual proteinuria after at least 6 months of traditional treatment at renal biopsy.

Valuable	RASI group (*n* = 183)	HCQ + RASI group (*n* = 59)	RASI + P ± IM group (*n* = 145)	HCQ + RASI + P ± IM group (*n* = 38)	*P*_1_	*P*_2_
Age (year)	31.0 (27.0, 43.0)	30.0 (26.0, 40.0)	34.0 (26.5, 44.0)	31.5 (25.8, 39.5)	0.237	0.426
Sex (Male/Female)	70/113	23/36	64/81	17/21	0.920	0.947
Follow-up time (month)	5.9 (5.6, 6.3)	6.1 (5.6, 6.5)	6.0 (5.7, 6.3)	6 (5.6, 6.3)	0.122	0.541
BMI (kg/m^2^)	22.7 (20.6, 25.6)	22.7 (20.5, 25)	23.2 (20.6, 25.6)	22.1 (19.9, 24.5)	0.997	0.096
MAP (mmHg)	98 (88.3, 106.3)	99.3 (88.3, 105.3)	96.3 (90.3, 108.3)	97.8 (88.3, 107.1)	0.503	0.761
Hb (g/L)	132.0 ± 23.0	130.7 ± 18.2	130.3 ± 24.6	129.9 ± 17.5	0.689	0.922
BUN (mmol/L)	5.6 (4.6, 6.5)	6.0 (4.6, 7.9)	6.1 (4.8, 8.3)	6.5 (4.7, 8.3)	0.158	0.964
UA (μmol/L)	363.3 ± 119.1	381.0 ± 125.2	385.1 ± 117.2	386.5 ± 135.1	0.338	0.951
Alb (g/L)	39.4 (35.3, 42.7)	40.5 (35.6, 42.7)	36.4 (30.7, 40.8)	36.8 (33.1, 42.3)	0.597	0.259
24-h Upro (g/24h)	1.3 (0.8, 2.0)	1.8 (1.0, 2.7)	2.5 (1.4, 3.7)	2.6 (1.2, 3.8)	0.056	0.794
Scr (μmol/L)	78.2 (58.0, 100.8)	87.8 (64.6, 125.0)	94.4 (65.3, 118.8)	99.7 (67.9, 126.7)	0.069	0.521
eGFR (ml/min/1.73m^2^)	98.9 (68.5, 119.1)	81 (53.9, 114.4)	85.0 (56.7, 111.8)	75.7 (48.0, 111.3)	0.050	0.542
Mesangial hypercellularity, n (%)					0.384	0.258
M0	117 (63.9%)	34 (57.6%)	69 (47.6%)	22 (57.9%)		
M1	66 (36.1%)	25 (42.4%)	76 (52.4%)	16 (42.1%)		
Endocapillary hypercellularity, n (%)					0.503	0.645
E0	138 (75.4%)	47 (79.7%)	112 (77.2%)	28 (73.7%)		
E1	45 (24.6%)	12 (20.3%)	33 (22.8%)	10 (26.3%)		
Segmental glomerulosclerosis, n (%)					0.118	0.304
S0	102 (55.7%)	26 (44.1%)	63 (43.4%)	13 (34.2%)		
S1	81 (44.3%)	33 (55.9%)	82 (56.6%)	25 (65.8%)		
Tubular atrophy/interstitial fibrosis, n (%)					0.207	0.246
T0	169 (92.3%)	54 (91.5%)	122 (84.1%)	28 (73.7%)		
T1	14 (7.7%)	4 (6.8%)	22 (15.2%)	10 (26.3%)		
T2	0 (0%)	1 (1.7%)	1 (0.7%)	0 (0%)		
Cellular/fibrocellular crescents, n (%)					0.923	0.140
C0	93 (50.8%)	29 (49.2%)	72 (49.7%)	24 (63.2%)		
C1	80 (43.7%)	26 (44.1%)	55 (37.9%)	13 (34.2%)		
C2	10 (5.5%)	4 (6.8%)	18 (12.4%)	1 (2.6%)		
Drug regimen before enrollment (%)					0.019*	0.667
RASI	100%	100%	99.3%	100%		
P	48.1%	89.8%	84.8%	94.7%		
IM	34.4%	52.5%	76.6%	65.8%		

*BMI, body mass index; MAP, mean arterial pressure; Hb, hemoglobin; BUN, blood urea nitrogen; UA, uric acid; Alb, albumin; 24-h Upro, 24-hour urine protein; Scr, serum creatinine; eGFR, estimated glomerular filtration rate; HCQ, hydroxychloroquine; P, corticosteroids; IM, immunosuppressives; Mesangial hypercellularity (M0/M1, <or equal to >50% of glomeruli with >4 mesangial cells/area); Endocapillary hypercellularity (E0/E1, absent/present); Segmental glomerulosclerosis (S0/S1, absent/present); Tubular atrophy/interstitial fibrosis (T0/T1/T2, <25%, 25–50%, >50%). Cellular/fibrocellular crescents (C0/C1/C2, absent or crescents in a least 1 but <25% of glomeruli or crescents in at least 25% of glomeruli). 1 represents the comparison between the RASI group and HCQ + RASI group; 2 represents the comparison between RASI + P ± IM group and HCQ + RASI + P ± IM group.*

*Represents statistical significance (p<0.05).

### Comparison of Clinical and Pathological Data at the Enrollment

Compare to patients not receiving HCQ, the residual proteinuria of patients receiving HCQ at the enrollment was significantly higher [1.10 (0.70, 1.44) g/24 h vs. 0.74 (0.51, 1.26) g/24 h, *p* < 0.0001], and their renal function was worse [99.30 (70.50, 140.95) μmol/L vs. 83.65 (63.20, 105.30) μmol/L, *p* = 0.001].

At the enrollment, the HCQ + RASI group had a higher level of residual proteinuria and a worse renal function than those in the RASI group, as shown in Table 2. Also, there were significant differences in patients with residual proteinuria <1 g/24 h and ≥1 g/24 h between the two groups [143 (78.1%) cases and 40 (21.9%) in the RASI group, respectively; 29 (49.2%) cases and 30 (50.8%) cases in HCQ + RASI group, respectively; *p* < 0.0001]. In addition, the renal function at the enrollment was worse in the HCQ + RASI + P ± IM group than that in the control group, but residual proteinuria levels were similar, as shown in Table 2. In RASI + P ± IM group, patients with residual proteinuria <1 g/24 h and ≥1 g/24 h were 70 (48.3%) cases and 75 (51.7%) cases, respectively. In HCQ + RASI + P ± IM group, patients with residual proteinuria <1 g/24 h and ≥1 g/24 h were 11 (28.9%) cases and 27 (71.1%) cases. The difference was statistically significant.

**TABLE 2:** The clinicopathological characteristics of IgAN patients with residual proteinuria after at least 6 months of traditional treatment at the enrollment.

Valuable	RASI group (*n* = 183)	HCQ + RASI group (*n* = 59)	RASI + P ± IM group (*n* = 145)	HCQ + RASI + P ± IM group (*n* = 38)	*P*_1_	*P*_2_
Hb (g/L)	132.0 (122.0, 146.0)	126.0 (116.0, 146.0)	133.0 (115.5, 148.0)	135.0 (121.5, 150.0)	0.265	0.623
UA (μmol/L)	380.1 ± 106.1	400.2 ± 118.9	406.6 ± 107.3	415.3 ± 74.6	0.321	0.618
Alb (g/L)	43.8 (41.1, 45.2)	42.7 (41.3, 44.8)	41.5 (38.4, 43.7)	41.3 (39.1, 43.8)	0.630	0.952
24-h Upro (g/24h)	0.7 (0.5, 0.9)	1.0 (0.7, 1.4)	1.0 (0.6, 1.8)	1.3 (0.9, 1.9)	<0.0001*	0.143
Scr (μmol/L)	78.0 (59.8, 99.3)	89.2 (69.9, 132.0)	100.7 (68.9, 117.0)	105.9 (78.4, 147.3)	0.001*	0.046*
eGFR (ml/min/1.73m^2^)	97.4 (73.5, 116.6)	67.4 (46.5, 102.6)	83.4 (56.8, 108.5)	69.4 (41.6, 98.3)	<0.0001*	0.043*
Proteinuria level grouping, n (%)					<0.0001*	0.033*
<1 g/24h	143 (78.1%)	29 (49.2%)	70 (48.3%)	11 (28.9%)		
≥ 1 g/24h	40 (21.9%)	30 (50.8%)	75 (51.7%)	27 (71.1%)		

*Hb, hemoglobin; BUN, blood urea nitrogen; UA, uric acid; Alb, albumin; 24-h Upro, 24-hour urine protein; Scr, serum creatinine; eGFR, estimated glomerular filtration rate; 1 represents the comparison between RASI group and HCQ + RASI group; 2 represents the comparison between RASI + P ± IM group and HCQ + RASI + P ± IM group.*

*Represents statistical significance (p<0.05).

### Matching Factors With Different Distribution by Propensity Score Matching

**FIGURE 2:**
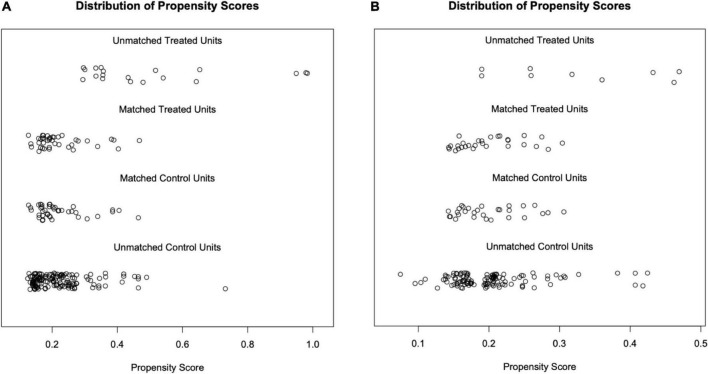
Distribution of propensity scores. Each circle represents an individual, and its corresponding value on the *x*-axis represents its propensity score. **(A)** After matching, the propensity scores of the RASI group were mainly distributed from 0.10 to 0.50, as well as the HCQ + RASI group. **(B)** After matching, the propensity scores of both groups, the RASI + P ± IM group and HCQ + RASI + P ± IM group, were mainly distributed from 0.15 to 0.35.

After matching factors (residual proteinuria and serum creatinine at enrollment) with different distribution by propensity score matching (PSM), there were 40 patients in the RASI group and HCQ + RASI group, respectively, and 29 patients in RASI + P ± IM group and HCQ + RASI + P ± IM group, respectively. There were no statistically significant differences in 24-h Upro, Scr, eGFR, and IgAN-MESTC scores between the first two groups, nor between the latter two groups (shown in Table 3). We plotted the distribution of propensity scores (shown in Figure 2) and found that propensity scores were consistent, suggesting that the population characteristics were consistent.

**TABLE 3:** The clinicopathological characteristics of IgAN patients at the time of enrollment after matching.

Valuable	RASI group (*n* = 40)	HCQ + RASI group (*n* = 40)	RASI + P ± IM group (*n* = 29)	HCQ + RASI + P ± IM group (*n* = 29)	*P*_1_	*P*_2_
Hb (g/L)	128.6 ± 21.6	127.5 ± 16.7	129.4 ± 23.4	134.1 ± 21.0	0.883	0.583
BUN (mmol/L)	6.3 ± 1.7	6.1 ± 2.3	8.2 ± 3.1	7.1 ± 2.6	0.778	0.204
UA (μmol/L)	361.3 ± 131.9	358.4 ± 101.4	424.4 ± 99.5	418.9 ± 78.6	0.930	0.844
Alb (g/L)	42.9 ± 4.4	42.9 ± 3.1	41.0 ± 5.7	40.7 ± 2.5	0.937	0.815
24 h Upro (g/24h)	0.7 (0.4, 1.0)	0.8 (0.6, 1.2)	1.06 (0.54, 2.11)	1.29 (1.02, 2.18)	0.063	0.222
Scr (μmol/L)	76.0 (65.3, 95.8)	74.2 (63.7, 99.8)	100.7 (64.3, 120.4)	90.1 (67.1, 128.1)	0.795	0.895
eGFR (ml/min/1.73m^2^)	97.4 (77.3, 119.9)	91.1 (63.3, 119.5)	76.2 (60.0, 102.2)	74.6 (51.0, 107.1)	0.523	0.680
Mesangial hypercellularity, n (%)					0.366	0.189
M0	25 (62.5%)	21 (52.5%)	12 (41.4%)	17 (58.6%)		
M1	15 (37.5%)	19 (47.5%)	17 (58.6%)	12 (41.4%)		
Endocapillary hypercellularity, n (%)					0.204	0.764
E0	27 (67.5%)	32 (80.0%)	22 (75.9%)	21 (72.4%)		
E1	13 (32.5%)	8 (20.0%)	7 (24.1%)	8 (27.6%)		
Segmental glomerulosclerosis, n (%)					0.823	0.594
S0	21 (52.5%)	20 (50.0%)	13 (44.8%)	11 (37.9%)		
S1	19 (47.5%)	20 (50.0%)	16 (55.2%)	18 (62.1%)		
Tubular atrophy/interstitial fibrosis, n (%)					0.077	1.000
T0	36 (90.0%)	39 (97.5%)	24 (82.8%)	24 (82.8%)		
T1	4 (10.0%)	0 (0%)	5 (17.2%)	5 (17.2%)		
T2	0 (0%)	1 (2.5%)	-	-		
Cellular/fibrocellular crescents, n (%)					0.622	0.362
C0	19 (47.5%)	20 (50%)	16 (55.2%)	17 (58.6%)		
C1	19 (47.5%)	16 (40%)	9 (31.0%)	11 (37.9%)		
C2	2 (5%)	4 (10%)	4 (13.8%)	1 (3.4%)		

*Hb, hemoglobin; BUN, blood urea nitrogen; UA, uric acid; Alb, albumin; 24-h Upro, 24-hour urine protein; Scr, serum creatinine; eGFR, estimated glomerular filtration rate; 1 represents the comparison between RASI group and HCQ + RASI group; 2 represents the comparison between RASI + P ± IM group and HCQ + RASI + P ± IM group.*

### Primary Renal Outcome

**FIGURE 3:**
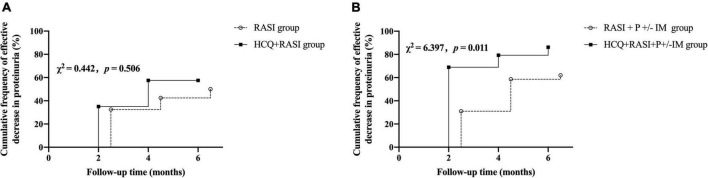
The cumulative frequency of patients with an effective decrease in residual proteinuria during the follow-up period. **(A)** The RASI group vs. the HCQ + RASI group. **(B)** The RASI + P ± IM group vs. the HCQ + RASI + P ± IM group.

At 6 months, the cumulative frequency of patients with an effective decrease in residual proteinuria was comparable between the RASI group and HCQ + RASI group but was significantly higher in the HCQ + RASI + P ± IM group than that in the control group (62.1% for patients with RASI + P ± IM vs. 86.2% for patients with HCQ + RASI + P ± IM, χ^2^ = 6.397, *p* = 0.011, shown in Figure 3).

### Secondary Renal Outcome

**FIGURE 4:**
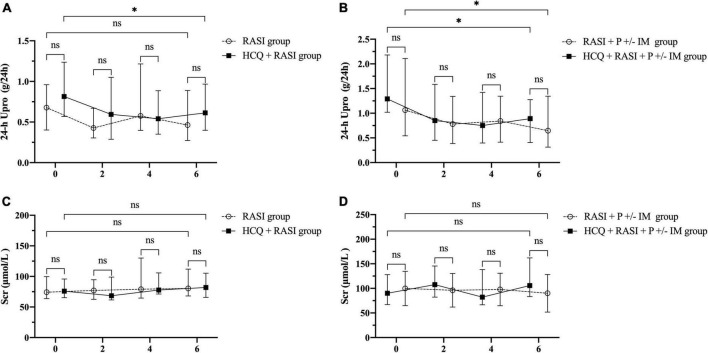
The residual proteinuria and renal function during the follow-up period. 0, 2, 4, and 6 indicate the time of enrollment, the 2nd, 4th, and 6th month, respectively. **(A)** The residual proteinuria in the RASI group vs. the HCQ + RASI group. **(B)** The residual proteinuria in the RASI + P ± IM group vs. the HCQ + RASI + P ± IM group. **(C)** Scr in the RASI group vs. the HCQ + RASI group. **(D)** Scr in the RASI + P ± IM group vs. the HCQ + RASI + P ± IM group. *Represents statistical significance (*p* < 0.05).

**FIGURE 5:**
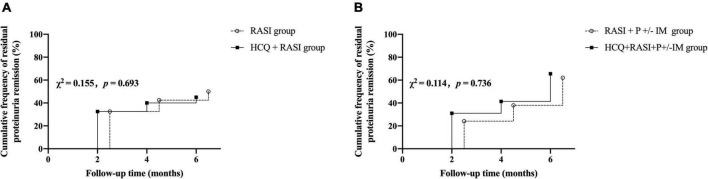
The cumulative frequency of residual proteinuria remission during the follow-up period. **(A)** The RASI group vs. the HCQ + RASI group. **(B)** The RASI + P ± IM group vs. the HCQ + RASI + P ± IM group.

Residual proteinuria levels, Δ24 h proteinuria quantification, the percent changes in proteinuria and Scr during follow-up were no statistical differences between the RASI group and the HCQ + RASI group (shown in Table 4). The level of residual proteinuria at the 6th month in the HCQ + RASI group was lower than that at the enrollment (*Z* = −3.299, *p* = 0.001), but no change was observed in the RASI group (*Z* = −1.156, *p* = 0.248), as shown in Figure 4. No significant change was observed in Scr before and after HCQ use in Figure 4. At the 6 months, the cumulative frequency of residual proteinuria remission in the HCQ + RASI group was 45.0% for patients treated with RASI-alone and 50.1% for patients treated with HCQ + RASI (χ^2^ = 0.155, *p* = 0.693, shown in Figure 5).

**TABLE 4:** Indicators related to residual proteinuria and renal function during follow-up of matched IgAN patients.

Valuable	RASI group (*n* = 40)	HCQ + RASI group (*n* = 40)	RASI + P ± IM group (*n* = 29)	HCQ + RASI + P ± IM group (*n* = 29)	*P*_1_	*P*_2_
Residual proteinuria (g/24h)						
0	0.68 (0.40, 0.96)	0.81 (0.57, 1.24)	1.06 (0.54, 2.11)	1.29 (1.02, 2.18)	0.063	0.222
2	0.43 (0.30, 0.67)	0.59 (0.29, 1.05)	0.78 (0.39, 1.34)	0.85 (0.45, 1.59)	0.266	0.597
4	0.58 (0.40, 1.22)	0.54 (0.35, 0.89)	0.84 (0.41, 1.34)	0.75 (0.40, 1.42)	0.611	0.949
6	0.46 (0.27, 0.89)	0.61 (0.40, 0.97)	0.65 (0.31, 1.35)	0.89 (0.41, 1.27)	0.337	0.319
**Δ**24 h Upro (g/24h)						
2	–0.31 (–0.49, –0.02)	–0.20 (–0.63, 0)	–0.24 (–0.83, 0.09)	–0.57 (–1.24, –0.22)	0.798	0.179
4	0.08 (–0.26, 0.45)	–0.26 (–0.55, 0.03)	–0.49 (–1.14, 0)	–0.40 (–0.84, –0.11)	0.037	0.666
6	–0.04 (–0.23, 0.10)	–0.26 (–0.51, 0.01)	–0.30 (–1.20, 0.05)	–0.45 (–0.97, 0.03)	0.087	0.887
Percent changes in proteinuria (%)						
2	–32.89 (–58.93, –5.26)	–20.94 (–63.27, 0)	–27.29 (–57.51, 9.19)	–46.97 (–59.90, –17.57)	0.735	0.309
4	5.37 (–45.55, 68.65)	–31.33 (–62.50, 2.31)	–49.01 (–59.91, 0.27)	–40.68 (–61.47, –7.91)	0.103	0.722
6	–5.02 (–54.84, 14.04)	–31.18 (–56.00, 0.91)	–45.13 (–75.29, 13.41)	–38.98 (–67.26, 2.69)	0.278	0.569
Scr (μmol/L)						
0	75.95 (65.25, 95.80)	74.20 (63.68, 99.83)	100.67 (64.25, 120.40)	90.10 (67.05, 128.10)	0.795	0.895
2	68.40 (61.60, 98.85)	77.00 (62.53, 94.60)	90.40 (70.60, 112.20)	107.50 (82.10, 145.55)	0.534	0.410
4	77.80 (71.18, 105.80)	79.00 (64.60, 130.00)	96.45 (76.83, 124.63)	82.10 (66.50, 138.30)	0.799	0.914
6	81.90 (65.70, 105.30)	80.40 (68.15, 111.90)	83.35 (60.40, 115.15)	105.70 (83.18, 162.05)	0.882	0.075
eGFR (ml/min/1.73m^2^)						
0	97.41 (77.31, 119.94)	91.07 (63.29, 119.54)	76.22 (60.01, 102.22)	74.60 (51.00, 107.05)	0.523	0.680
2	105.10 (64.64, 116.57)	98.30 (70.99, 120.27)	74.84 (52.81, 118.54)	72.10 (46.55, 90.00)	0.836	0.196
4	86.65 (72.41, 109.92)	82.82 (66.26, 119.47)	73.04 (59.41, 95.99)	77.88 (39.35, 100.85)	0.913	0.692
6	96.06 (61.33, 118.92)	84.78 (55.85, 121.77)	85.92 (65.36, 121.85)	62.95 (41.10, 99.58)	0.866	0.104

*Δ24 h Upro was calculated as the difference between the 24-hour urine protein quantification at a time point during the follow-up period and the time of enrollment. Scr, serum creatinine; eGFR, estimated glomerular filtration rate; 0, 2, 4, and 6 indicate the time of enrollment, the 2nd, 4th and 6th month, respectively. 1 represents the comparison between the RASI group and HCQ + RASI group; 2 represents the comparison between RASI + P ± IM group and HCQ + RASI + P ± IM group.*

There was no difference in residual proteinuria levels, Δ24 h proteinuria quantification, the percent changes in proteinuria and Scr during follow-up between the RASI + P ± IM group and HCQ + RASI + P ± IM group (shown in Table 4). HCQ + RASI + P ± IM group had a significant decrease in residual proteinuria level from 1.29 (1.02, 2.18) g/24 h at the enrollment time to 0.89 (0.41, 1.27) g/24 h at the 6th month [*Z* = –2.926, *p* = 0.003], as well as in the RASI + P ± IM group [*Z* = –2.343, *p* = 0.019]. However, Scr were comparable before and after HCQ use (shown in Figure 4). At the 6 months, the cumulative frequency of residual proteinuria remission was 62.1% for patients with RASI + P ± IM and 65.5% for patients with HCQ + RASI + P ± IM (χ^2^ = 0.114, *p* = 0.736, shown in Figure 5).

### Clinicopathological Indicators Associated With the Effective Reduction in Residual Proteinuria

Among the patients whose sequential treatment with RASI or HCQ + RASI, 76 (63.3%) patients reached the effective reduction frequency in residual proteinuria. There was no correlation between clinicopathological features and the effective reduction frequency in residual proteinuria. Univariate and multifactorial logistic regression analysis didn’t support the addition of HCQ treatment as an independent factor for a ≥30% decrease in residual proteinuria (shown in Table 5).

**TABLE 5:** Clinicopathological features associated with the effective reduction frequency in residual proteinuria in patients treated with RASI or HCQ + RASI.

Valuable	*r*	*P*	Univariate		Multivariat*e*	
			OR (95% CI)	*P*	OR (95% CI)	*P*
Sex	–0.022	0.850	0.91 (0.37, 2.28)	0.847	0.49 (0.12, 1.99)	0.321
Age	–0.016	0.886	0.99 (0.95, 1.03)	0.716	0.97 (0.91, 1.03)	0.362
BMI	–0.106	0.376	0.94 (0.83, 1.07)	0.347		
MAP at the biopsy	0.228	0.052	1.04 (0.99, 1.08)	0.104	1.05 (0.99, 1.11)	0.149
HGB at the biopsy	0.141	0.227	1.01 (0.99, 1.03)	0.223		
HGB at the enrollment	0.263	0.205	1.03 (0.98, 1.08)	0.261		
Upro at the biopsy	–0.014	0.899	1.10 (0.81, 1.50)	0.546	0.91 (0.62, 1.32)	0.613
Upro at the enrollment	0.152	0.178	1.93 (0.72, 5.16)	0.191	2.52 (0.67, 9.47)	0.172
Scr at the biopsy	0.015	0.897	1.00 (0.99, 1.01)	0.861	1.00 (0.97, 1.04)	0.813
Scr at the enrollment	–0.031	0.782	0.99 (0.98, 1.01)	0.408	0.97 (0.94, 1.01)	0.138
Mesangial hypercellularity	–0.014	0.902	–	–		
M0			reference	1		
M1			0.95 (0.39, 2.30)	0.901		
Endocapillary hypercellularity	–0.187	0.096	–	–		
E0			reference	1		
E1			0.42 (0.15, 1.17)	0.098		
Segmental glomerulosclerosis	0.203	0.072	–	–		
S0			reference	1	reference	1
S1			2.28 (0.93, 5.61)	0.072	4.73 (1.35, 16.64)	0.015*
Tubular atrophy/interstitial fibrosis	0.035	0.758	–	–		
T0			reference	1		
T1			1.31 (0.21, 8.31)	0.773		
T2						
Cellular/fibrocellular crescents	0.038	0.740	–	–		
C0			reference	1		
C1			1.27 (0.51, 3.17)	0.614		
C2			0.95 (0.17, 5.30)	0.953		
Drug regimen before the enrollment						
RASI	–	–	–	–		
P	–0.122	0.281	0.57 (0.21, 1.57)	0.277	0.25 (0.05, 1.21)	0.085
IM	0.217	0.053	2.48 (0.98, 6.24)	0.055	1.90 (0.53, 6.85)	0.328
Addition of HCQ	0.075	0.507	1.35 (0.56, 3.27)	0.502	2.12 (0.63, 7.17)	0.227

*MAP, mean arterial pressure; Hb, hemoglobin; Scr, serum creatinine; HCQ, hydroxychloroquine; P, corticosteroids; IM, immunosuppressives; Mesangial hypercellularity (M0/M1, <or equal to >50% of glomeruli with >4 mesangial cells/area); Endocapillary hypercellularity (E0/E1, absent/present); Segmental glomerulosclerosis (S0/S1, absent/present); Tubular atrophy/interstitial fibrosis (T0/T1/T2, <25%, 25–50%, >50%). Cellular/fibrocellular crescents (C0/C1/C2, absent or crescents in a least 1 but <25% of glomeruli or crescents in at least 25% of glomeruli).*

*Represents statistical significance (p< 0.05).

Among the patients whose sequential treatment with RASI + P ± IM or HCQ + RASI + P ± IM, 43 (74.1%) patients reached the effective reduction frequency in residual proteinuria. MAP at the biopsy and addition of HCQ were associated with an effective decrease in residual proteinuria. Univariate and multifactorial logistic regression analysis showed that males, age, MAP at the biopsy, corticosteroids use before the enrollment and the addition of HCQ treatment were independent factors for a ≥30% decrease in residual proteinuria (shown in Table 6).

**TABLE 6:** Clinicopathological features associated with the effective reduction frequency in residual proteinuria in patients treated with RASI + P ± IM or HCQ + RASI + P ± IM.

Valuable	*r*	*P*	Univariate		Multivariat*e*	
			OR (95% CI)	*P*	OR (95% CI)	*P*
Sex	0.246	0.063	3.11 (0.92, 10.46)	0.067	20.40 (1.12, 371.91)	0.042*
Age	–0.212	0.110	0.96 (0.91, 1.01)	0.113	0.87 (0.78, 0.98)	0.016*
BMI	–0.178	0.182	0.89 (0.74, 1.07)	0.229		
MAP at the biopsy	–0.373	0.004*	0.94 (0.89, 0.99)	0.013	0.92 (0.85, 1.000)	0.046*
HGB at the biopsy	–0.125	0.369	0.98 (0.95, 1.02)	0.293		
HGB at the enrollment	0.014	0.941	1.00 (0.96, 1.04)	0.942		
Upro at the biopsy	0.060	0.655	0.98 (0.71, 1.36)	0.916	1.63 (0.89, 3.00)	0.114
Upro at the enrollment	0.232	0.080	1.22 (0.72, 2.07)	0.467	1.53 (0.61, 3.82)	0.368
Cr at the biopsy	–0.107	0.430	1.00 (0.98, 1.01)	0.525	1.04 (0.98, 1.09)	0.207
Cr at the enrollment	–0.126	0.347	0.99 (0.98, 1.01)	0.435	0.96 (0.91, 1.01)	0.126
Mesangial hypercellularity	0.039	0.769	–	–		
M0	–	–	reference	1		
M1	–	–	1.20 (0.37, 3.89)	0.764		
Endocapillary hypercellularity	–0.191	0.152	–	–		
E0	–	–	reference	1		
E1	–	–	0.40 (0.11, 1.41)	0.153		
Segmental glomerulosclerosis	–0.096	0.471	–	–		
S0	–	–	reference	1		
S1	–	–	0.63 (0.18, 2.16)	0.464		
Tubular atrophy/interstitial fibrosis	0.061	0.649	–	–		
T0	–	–	reference	1		
T1	–	–	1.49 (0.28, 7.93)	0.643		
T2	–	–	–	–		
Cellular/fibrocellular crescents	0.076	0.570	–	–		
C0	–	–	reference	1		
C1	–	–	1.19 (0.36, 3.93)	0.778		
C2	–	–				
Drug regimen before the enrollment						
RASI	–	–	–	–	–	
P	0.239	0.070	5.13 (0.77, 34.31)	0.092	182.45 (2.52, 13198.89)	0.017*
IM	0.076	0.571	1.46 (0.41, 5.20)	0.564	4.37 (0.33, 58.31)	0.264
Addition of HCQ	0.276	0.036*	3.82 (1.05, 13.94)	0.043*	36.15 (2.082, 627.58)	0.014*

*MAP, mean arterial pressure; Hb, hemoglobin; Scr, serum creatinine; HCQ, hydroxychloroquine; P, corticosteroids; IM, immunosuppressives; Mesangial hypercellularity (M0/M1, <or equal to >50% of glomeruli with >4 mesangial cells/area); Endocapillary hypercellularity (E0/E1, absent/present); Segmental glomerulosclerosis (S0/S1, absent/present); Tubular atrophy/interstitial fibrosis (T0/T1/T2, <25%, 25–50%, >50%). Cellular/fibrocellular crescents (C0/C1/C2, absent or crescents in a least 1 but <25% of glomeruli or crescents in at least 25% of glomeruli).*

*Represents statistical significance (p<0.05).

### Adverse Events

All patients underwent an ophthalmologic examination before HCQ treatment, and the results did not show any abnormalities. And there were no serious adverse events observed during the follow-up period.

## Discussion

Yang first reported the anti-urinary protein effect of HCQ in IgAN. Subsequently, a retrospective PSM cohort study found that the cumulative effective rate of proteinuria decline was significantly higher in the HCQ group than that in the control group and that HCQ treatment was an independent factor for ≥30% decline in proteinuria in IgAN (17). Another study found that HCQ combined with RASI for 6 months resulted in a 50% reduction in proteinuria, while RASI treatment alone resulted in only a 14.8% further reduction in proteinuria (19). Similarly, our previous retrospective study found that the rate of decline in residual proteinuria at the 6th month in patients with HCQ + RASI + P/IM regimen was significantly higher than that in patients with RASI + P/IM regimen [−36.6 (−67.3, −9.1)% vs. −15.5 (−48.3, 17.2)%, *p* = 0.034] (24). In this study, we found that the addition of HCQ may result in more IgAN patients achieving an effective rate of decrease in residual proteinuria compared to patients with RASI + P ± IM sequential therapy and also was an independent factor for the reduction of residual proteinuria ≥30% in IgAN patients. A retrospective study with similar results to our study reported that HCQ had an anti-protein effect comparable to that of corticosteroids and might be an alternative or supplement to P and IM therapy (18).

Interestingly, except RASI + P ± IM group, we observed that the decrease in residual proteinuria after the addition of HCQ was more significant in the first 2 months and stabilized in the last 4 months, which might be attributed to the short-term after-effects of corticosteroids and immunosuppressive drugs. Meanwhile, we observed that HCQ had no significant effect on short-term renal function in CKD patients. Thus, longer-term follow-up observation is needed.

This study was a two-center retrospective study and small sample size. Due to the limitations of retrospective studies, patients with worse renal function and higher proteinuria received HCQ, which inevitably led to selective bias. Also, none of the patients in our study were monitored for HCQ drug concentrations during the follow-up period. Therefore, further multi-center, large sample randomized controlled trials are needed to further validate the long-term clinical efficacy and safety of HCQ in patients with IgAN.

## Data Availability Statement

The original contributions presented in this study are included in this article/supplementary material, further inquiries can be directed to the corresponding authors.

## Ethics Statement

This study was approved by the Ethics Committee of Sichuan Provincial People’s Hospital and Ethics Committee of Ningbo First Hospital. Written informed consent from the patients was not required to participate in this study in accordance with the national legislation and the institutional requirements.

## Author Contributions

ML collected clinical data, performed data analysis, data interpretation, study design, statistical analyses, and manuscript writing. XB collected clinical data, analyzed the data, and interpreted the data. GL and LW conceived the study, supervised experiments, interpreted data, and wrote the manuscript. All authors contributed to the article and approved the submitted version.

## Conflict of Interest

The authors declare that the research was conducted in the absence of any commercial or financial relationships that could be construed as a potential conflict of interest.

## Publisher’s Note

All claims expressed in this article are solely those of the authors and do not necessarily represent those of their affiliated organizations, or those of the publisher, the editors and the reviewers. Any product that may be evaluated in this article, or claim that may be made by its manufacturer, is not guaranteed or endorsed by the publisher.

## References

[1] Wyatt RJ Julian BA. IgA nephropathy. *N Engl J Med.* (2013) 368:2402–14. 23782179 10.1056/NEJMra1206793

[2] Hou JH Zhu HX Zhou ML Le WB Zeng CH Liang SS Changes in the spectrum of kidney diseases: an analysis of 40,759 biopsy-proven cases from 2003 to 2014 in China. *Kidney Dis (Basel).* (2018) 4:10–9. 10.1159/000484717 29594138 PMC5848489

[3] Le W Liang S Hu Y Deng K Bao H Zeng C Long-term renal survival and related risk factors in patients with IgA nephropathy: results from a cohort of 1155 cases in a Chinese adult population. *Nephrol Dial Transplant.* (2012) 27:1479–85. 10.1093/ndt/gfr527 21965586

[4] Goto M Wakai K Kawamura T Ando M Endoh M Tomino Y. A scoring system to predict renal outcome in IgA nephropathy: a nationwide 10-year prospective cohort study. *Nephrol Dial Transplant.* (2009) 24:3068–74. 10.1093/ndt/gfp273 19515800 PMC2747499

[5] Reich HN Troyanov S Scholey JW Cattran DC Toronto Glomerulonephritis Registry. Remission of proteinuria improves prognosis in IgA nephropathy. *J Am Soc Nephrol.* (2007) 18:3177–83. 10.1681/ASN.2007050526 17978307

[6] Kidney Disease: Improving Global Outcomes Glomerular Diseases Work Group. KDIGO 2021 clinical practice guideline for the management of glomerular diseases. *Kidney Int.* (2021) 100:S1–276. 10.1016/j.kint.2021.05.021 34556256

[7] Canney M Barbour SJ Zheng Y Coppo R Zhang H Liu ZH Quantifying duration of proteinuria remission and association with clinical outcome in IgA nephropathy. *J Am Soc Nephrol.* (2021) 32:436–47. 10.1681/ASN.2020030349 33514642 PMC8054888

[8] Thompson A Carroll K Inker LA Floege J Perkovic V Boyer-Suavet S Proteinuria reduction as a surrogate end point in trials of IgA nephropathy. *Clin J Am Soc Nephrol.* (2019) 14:469–81. 10.2215/CJN.08600718 30635299 PMC6419287

[9] Yuan Y Che X Ni Z Zhong Y Qi Y Shao X Association of relapse with renal outcomes under the current therapy regimen for IgA nephropathy: a multi-center study. *PLoS One.* (2015) 10:e0137870. 10.1371/journal.pone.0137870 26371477 PMC4570760

[10] Lv J Xu D Perkovic V Ma X Johnson DW Woodward M Corticosteroid therapy in IgA nephropathy. *J Am Soc Nephrol.* (2012) 23:1108–16. 22539830 10.1681/ASN.2011111112 PMC3358763

[11] Usui J Yamagata K Kai H Outeki T Yamamoto S Muro K Heterogeneity of prognosis in adult IgA nephropathy, especially with mild proteinuria or mild histological features. *Intern Med.* (2001) 40:697–702. 10.2169/internalmedicine.40.697 11518105

[12] Shen P He L Huang D. Clinical course and prognostic factors of clinical early IgA nephropathy. *Neth J Med.* (2008) 66:242–7. 18689907

[13] Chen S Yin Q Ren S Zhong X Wang W Li G A comparison of the effectiveness of cyclophosphamide, leflunomide, corticosteroids, or conservative management alone in patients with IgA nephropathy: a retrospective observational study. *Sci Rep.* (2018) 8:13662. 10.1038/s41598-018-31727-5 30209279 PMC6135814

[14] Blaas SH Stieber-Gunckel M Falk W Obermeier F Rogler G. CpG-oligodeoxynucleotides stimulate immunoglobulin A secretion in intestinal mucosal B cells. *Clin Exp Immunol.* (2009) 155:534–40. 10.1111/j.1365-2249.2008.03855.x 19220839 PMC2669530

[15] Makita Y Suzuki H Kano T Takahata A Julian BA Novak J TLR9 activation induces aberrant IgA glycosylation via APRIL- and IL-6-mediated pathways in IgA nephropathy. *Kidney Int.* (2019) 97:340–9. 10.1016/j.kint.2019.08.022 31748116 PMC7372907

[16] Gao R Wu W Wen Y Li X. Hydroxychloroquine alleviates persistent proteinuria in IgA nephropathy. *Int Urol Nephrol.* (2017) 49:1233–41. 10.1007/s11255-017-1574-2 28349446

[17] Yang YZ Liu LJ Shi SF Wang JW Chen YQ Lv JC Effects of hydroxychloroquine on proteinuria in immunoglobulin A nephropathy. *Am J Nephrol.* (2018) 47:145–52. 10.1159/000487330 29502121

[18] Yang YZ Chen P Liu LJ Cai QQ Shi SF Chen YQ Comparison of the effects of hydroxychloroquine and corticosteroid treatment on proteinuria in IgA nephropathy: a case-control study. *BMC Nephrol.* (2019) 20:297. 10.1186/s12882-019-1488-6 31382914 PMC6683466

[19] Liu LJ Yang YZ Shi SF Bao YF Yang C Zhu SN Effects of hydroxychloroquine on proteinuria in IgA nephropathy: a randomized controlled trial. *Am J Kidney Dis.* (2019) 74:15–22. 10.1053/j.ajkd.2019.01.026 30922594

[20] Tang C Lv JC Shi SF Chen YQ Liu LJ Zhang H. Long-term safety and efficacy of hydroxychloroquine in patients with IgA nephropathy: a single-center experience. *J Nephrol.* (2022) 35:429–40. 10.1007/s40620-021-00988-1 33591553

[21] Trimarchi H Barratt J Cattran DC Cook HT Coppo R Haas M Oxford classification of IgA nephropathy 2016: an update from the IgA nephropathy classification working group. *Kidney Int.* (2017) 91:1014–21. 10.1016/j.kint.2017.02.003 28341274

[22] Inker LA Schmid CH Tighiouart H Eckfeldt JH Feldman HI Greene T Estimating glomerular filtration rate from serum creatinine and cystatin C. *N Engl J Med.* (2012) 367:20–9. 22762315 10.1056/NEJMoa1114248 PMC4398023

[23] Lv J Zhang H Wong MG Jardine MJ Hladunewich M Jha V Effect of oral methylprednisolone on clinical outcomes in patients with IgA nephropathy: the TESTING randomized clinical trial. *JAMA.* (2017) 318:432–42. 10.1001/jama.2017.9362 28763548 PMC5817603

[24] Liu MJ Li GS. The effect of hydroxychloroquine combination therapy on residual proteinuria in IgA nephropathy. *Prac J Clin Med.* (2021) 18:142–5.

